# Biochemical Changes in Blood of Patients with Duchenne Muscular Dystrophy Treated with Granulocyte-Colony Stimulating Factor

**DOI:** 10.1155/2019/4789101

**Published:** 2019-03-13

**Authors:** Dorota Sienkiewicz, Wojciech Kułak, Grażyna Paszko-Patej, Bożena Okurowska-Zawada, Jerzy Sienkiewicz, Piotr Kułak

**Affiliations:** ^1^Department of Pediatric Rehabilitation, Medical University of Bialystok, Bialystok, Poland; ^2^Bialystok Technical University, Bialystok, Poland; ^3^Department of Pediatric Radiology, Medical University of Bialystok, Bialystok, Poland

## Abstract

**Introduction:**

In addition to the “gold standard” of therapy—steroids and gene therapy–there are experimental trials using granulocyte-colony stimulating factor (G-CSF) for patients with Duchenne muscular dystrophy (DMD). The aim of this study was to present the biochemical changes in blood after repeating cycles of granulocyte-colony stimulating factor G-CSF therapy in children with DMD.

**Materials and Methods:**

Nineteen patients, aged 5 to 15 years, with diagnosed DMD confirmed by genetic tests, participated; nine were in wheelchairs, and ten were mobile and independent. Patients had a clinical assessment and laboratory tests to evaluate hematological parameters and biochemistry. G-CSF (5*μ*g/kg/day) was given subcutaneously for five days during five nonconsecutive months over the course of a year.

**Results:**

We found a significant elevation of white blood cells, and the level of leucocytes returned to norm after each cycle. No signs of any inflammatory process were found by monitoring C-reactive protein. We did not detect significant changes in red blood cells, hemoglobin, and platelet levels or coagulation parameters. We found a significant elevation of uric acid, with normalization after finishing each treatment cycle. A significant decrease of the mean value activity of aspartate transaminase (AST) and alanine transaminase (ALT) of the G-CSF treatment was noted. After each five days of therapy, the level of cholesterol was significantly lowered. Also, glucose concentration significantly decreased after the fourth cycle.

**Conclusions:**

G-SCF decreased the aminotransferases activity, cholesterol level, and glucose level in patients with DMD, which may be important for patients with DMD and metabolic syndrome.

## 1. Introduction

Granulocyte-colony stimulating factor (G-CSF) is a glycoprotein produced by endothelium, macrophages, and lymphocytes [1]. G-CSF stimulates the production of granulocyte progenitors and granulocyte maturation, phagocytosis, and superoxide production. Currently, G-CSF is widely used as a therapy for neutropenia induced by chemotherapy [2]. In addition, G-CSF is a strong stimulator of stem cells [3]. Additionally, G-CSF affects the brain's progenitor and nerve cells [4]. It has also been demonstrated that G-CSF affects metabolism, like leptin in animals and humans [5–8].

Triglycerides and free fatty acids were decreased in the experimental study of hepatic steatosis rats after G-CSF treatment [5]. G-CSF has a chemical structure similar to leptin and ciliary neurotrophic factor (CNTF). It is known that leptin and CNTF play significant roles in energy homeostasis and obesity [6].

In healthy donors who received G-CSF in different doses per day, changes in serum chemistries were observed [7]. Significant increases in aminotransferases and sodium and decreases in glucose and potassium bilirubin were found.

In a 2018 study, Hatfield et al. [8] evaluated the profile of 239 metabolites in stem cell donors before and after administration of G-CSF and found increased levels of long-chain fatty acids and polyunsaturated fatty acids. Moreover, lower concentrations of phospholipids, lysolipids, and sphingolipids were detected. Decreased levels of methionine, tryptophan, and valine were found. Also, carbohydrates and energy metabolism changes were detected.

In our previous prospective, nonrandomized clinical trial, we assessed the efficacy and safety of G-CSF treatment in patients with DMD [9]. Each participant received G-CSF (5 *μ*g/kg/body/day) subcutaneously for five consecutive days during the 1st, 2nd, 3rd, 6th, and 12th month. We found an increase of physical activity and muscle strength in patients with DMD treated with G-CSF during one year of therapy [9]. Also, we found significant decrease in the activity of the muscle enzyme creatine kinase after nearly every cycle of treatment. However, we did not present detailed impacts of G-CSF on biochemistry in children with DMD.

DMD is a one of the more frequent genetic disorders in children, characterized by progressive muscle weakness and wasting [10, 11]. The condition is caused by a deficiency of dystrophin, an important protein of cytoskeleton in the skeletal and cardiac muscles [12, 13]. The symptoms, due to muscle fragility, contraction-induced damage, necrosis, inflammation, and impaired angiogenesis [14–16], are motor retardation, cardiomyopathy, and respiratory failure [17].

G-CSF was tested in the animal model of DMD, the mdx mouse [18]. It was noted that treated mdx mice had a higher number of normal muscle fibres compared with untreated mdx mice. Also, a reduction in inflammation in the muscles was observed. Hayashiji et al. [19] showed that granulocyte-colony-stimulating factor receptor (G-CSFR) is expressed in activated satellite cells [19]. G-CSF positively affected the satellite cell population during multiple stages of differentiation in ex vivo cultured fibres. The authors suggested that G-CSF could be important in developing an effective therapy for DMD based on the regeneration of myocytes.

The “gold standard” of DMD therapy is steroids, which slow the course of the illness [20]. Contemporary, alternative therapeutic approaches for patients with muscular dystrophies use gene therapy, cellular therapy, and G-CSF [9, 21].

It is worth emphasizing that most children and adolescents with DMD have symptoms of metabolic syndrome. Hypertriglyceridemia is prevalent in 46% of children and adolescents with DMD. For example, the prevalence of metabolic syndrome varies between 7% and 24% [22].

This study aimed to present the biochemical changes in blood after repeating cycles of G-CSF treatment in patients with DMD.

## 2. Materials and Methods

### 2.1. Study Design

A retrospective, nonrandomized clinical trial evaluated the safety of G-CSF therapy in patients with DMD: biochemical changes in the blood after repeating cycles of treatment.

### 2.2. Participants

The following inclusion criteria were used in this study: children and adolescents aged 5-15 years with diagnosed DMD; the disease had to be confirmed by genetic test or biopsy. Nineteen subjects were included in the study. Fourteen patients took steroids. Nine children used wheelchairs and ten were mobile. Details are described in our previous article (Sienkiewicz et al. 2017).

### 2.3. Assessment

Patients were clinically monitored by neurological assessment. Laboratory tests included full blood count and biochemistry (cholesterol, CRP, glucose, electrolytes, coagulation system, and uric acid) made by laboratory at the Medical University Children Hospital, Bialystok. Blood was drawn before G-CSF application and on the fifth day of each treatment cycle.

### 2.4. G-CSF Application

Granulocyte-colony stimulating factor (5*μ*g/kg/body/day) was given subcutaneously for five days during the first, second, third, sixth, and twelfth month during hospitalisation. All patients received rehabilitation procedures.

### 2.5. Ethics

The bioethics committee of the Medical University of Bialystok, Poland, approved this study (R-I-002/375/2013). The parents and patients gave written informed consent to participate in the study before participation. The clinical trial was registered at the website Clinicaltrials.gov (NCT02814110).

### 2.6. Statistics

All data and statistical analyses were performed using SPSS statistical software program (version 15.0). Paired* t*-test was used to compare the difference over time. The results were considered significant at p<0.05.

## 3. Results

A total of 19 children and adolescents, boys aged 5-15 years (9.4±2.6), were treated with G-CSF. Sixteen participants (84.2%) completed the study, obtaining five cycles of five days' treatment (G-CSF in dose 5*μ*g/kg/day).

Blood samples were collected before and after each cycle of therapy. We found a significant increase (p<0.001) in the white blood cell count after each cycle of G-CSF, but no changes in the red blood cell, hemoglobin, platelet, and C-reactive protein levels. The level of leucocytes returned to normal after each cycle. Details are presented in Tables 1 and 2.

The coagulation system was monitored by prothrombin time, prothrombin index, kaolin-cephalin time and fibrinogen assay. There was no deterioration in these parameters (Table 3).

As shown in Table 4, we detected a significant increase in uric acid (p<0.001) in the patients during G-CSF therapy that decreased to a normal level after each cycle (Table 4).

We found decreases in aminotransferases activity and cholesterol level. We observed significantly lower AST activity after the first, second, third (each p=0.001), and fifth (p=0.023) cycle treatments. ALT activity decreased after the first (p=0.011) and third (p=0.006) cycles of G-CSF treatment. After each cycle, we observed significant (p<0.001) lowering of the total cholesterol level. These results are presented in Table 5.

We also found a significant decrease in glucose level after fourth cycle treatment. Details are shown in Table 6.

## 4. Discussion

In our previous article, we described an increase in physical activity and muscle strength in our study group patients treated with G-CSF during one year of therapy. As to laboratory parameters, we found a statistically significant decrease in the activity of muscle enzymes creatine kinase after each cycle of treatment [9].

In the present study, we did not detect significant changes in red blood cell, hemoglobin and platelet levels, or coagulation parameters. We found a significant elevation of uric acid, with normalization after finishing each cycle of treatment with G-CSF. We also observed decreased aminotransferases activity, cholesterol level, and glucose level in patients with DMD, which may be important for patients with DMD and metabolic syndrome.

Our findings are in agreement with earlier reports [8, 23]. In 40 healthy subjects who received filgrastim to prepare for apheresis, increases in neutrophil and lymphocyte counts, alkaline phosphatase and lactate dehydrogenase levels, and changes in serum electrolytes and uric acid were found. Laboratory changes subsided within a week of drug administration.

Possible mechanisms of the general effect of G-CSF on various liver diseases have been suggested. Stem cells of the bone marrow may accelerate the regeneration of damaged liver cells by differentiation or paracrine effect [24]. Additionally, G-CSF can act on liver cells via G-CSF receptors [25].

In an animal model of atherosclerosis, G-CSF reduced atherosclerotic plaque formation in cholesterol-fed rabbits [26]. Administration of G-CSF appears to lower total serum cholesterol without significantly affecting the relative proportions of lipoproteins [27]. In patients who received granulocyte-macrophage-colony-stimulating factor (GM-CSF), a decrease in serum cholesterol concentration was observed [28]. Serum cholesterol levels decreased by approximately 40%. G-CSF administration in healthy subjects decreased systemic levels of fatty acid metabolites involved in hydrolysis (phospholipid metabolism and lysolipids), whereas long/medium-chain fatty acids generally increased [8].

It is known that, in obese subjects, chronic inflammation is observed, with increased levels of proinflammatory cytokines, including IL-6 and tumor necrosis factor-n (TNF*α*), which increase insulin resistance [29–31]. However, G-CSF decreases activation of these cytokines and TNF*α* and decreases the harmful effects of inflammatory reactions [32].

Leptin controls glucose metabolism regardless of the impact on energy balance [33]. Thus, the decreased glucose level in our DMD patients may be explained by the leptin­like effect of G-CSF treatment.

We further suggest that our results, described above, are related to potential hepatic regeneration proprieties of G-CSF and stem cells. Stem cells repopulate the liver and differentiate into hepatic cells [34]. In animal studies, G-CSF treatment significantly improved survival and liver histology by stimulating endogenous repair mechanisms [23, 30, 31]. Also, after G-CSF administration to patients with end-stage liver disease, liver function enzymes remained stable [24].

## 5. Conclusions

In summary, our study shows changes in laboratory parameters, biochemical and hematological, in children with DMD during repeated cycles of treatment with G-CSF (5*μ*g/kg/day, 5 days). Statistically significant effects applied to (1) the increase of white blood count level after each treatment cycle, with following normalization and no change in CRP level, (2) elevation of uric acid and following normalization during therapy, and (3) diminution of AST, ALT activity and cholesterol level. We observed no alterations in hematological and coagulation system parameters. The G-CSF therapy was safe and well tolerated. These results are associated with the multidirectional, stimulating impacts of G-CSF on the organism, and show the safety of this type of treatment for patients with muscular dystrophies. However, because of the limited group size, the findings of the present study must be interpreted with great caution.

## Figures and Tables

**Table 1:** The hematological parameters in children and adolescents with Duchenne muscular dystrophy during treatment with granulocyte-colony stimulating factor.

Measurement		Red blood cells (4.5-5.5 10'6/*μ*l, normal range)			Hemoglobin (12.0 -15.5 g/dl, normal range)		
		Average	SD	P value	Average	SD	P value
Cycle 1	1	5.33	1.14	NS	13.82	0.83	NS
	2	5.11	0.33		13.83	0.83	

Cycle 2	1	5.10	0.34	NS	13.93	0.96	NS
	2	5.18	0.29		14.24	0.87	

Cycle 3	1	5.00	0.29	NS	13.57	0.96	NS
	2	5.04	0.30		13.7	1.08	

Cycle 4	1	5.12	0.36	NS	13.82	1.13	NS
	2	5.14	0.36		13.78	0.87	

Cycle 5	1	5.14	0.29	NS	13.86	0.86	NS
	2	5.09	0.31		13.83	1.00	

Measurement		Platelets (140.0-450.0 10'3/*μ*l, normal range)			Leucocyte (4.0-12.0 10'3/*μ*l, normal range)		
		Average	SD	P value	Average	SD	P value

Cycle 1	1	327.89	87.96	NS	7.06	2.05	<*0.001*
	2	322.00	63.33		29.53	13.23	

Cycle 2	1	381.88	106.84	NS	7.32	2.58	<*0.001*
	2	348.75	63.62		31.69	13.38	

Cycle 3	1	347.68	79.61	NS	6.66	1.89	<*0.001*
	2	354.74	77.61		32.66	12.17	

Cycle 4	1	327.11	69.76	NS	7.22	2.22	<*0.001*
	2	328.11	66.24		27.05	10.00	

Cycle 5	1	334.88	60.52	NS	7.16	2.51	<*0.001*
	2	319.00	70.77		24.23	10.59	

**Table 2:** C-reactive protein levels in children and adolescents with Duchenne muscular dystrophy during treatment with granulocyte-colony stimulating factor.

Measurement		CRP (0-5.0) normal range		P value
		Average	SD	
Cycle 1	1	0.61	0.69	NS
	2	0.41	0.42	

Cycle 2	1	0.97	2.09	NS
	2	1.09	2.34	

Cycle 3	1	0.63	0.61	NS
	2	1.17	1.46	

Cycle 4	1	0.63	0.69	NS
	2	0.60	0.51	

Cycle 5	1	0.50	0.51	NS
	2	0.50	0.55	

CRP, C-reactive protein; NS, nonsignificant.

**Table 3:** The parameters of coagulation system in children and adolescents with Duchenne muscular dystrophy during treatment with granulocyte-colony stimulating factor.

Measurement		Prothrombin time (10.4-14sec)		P	Prothrombin index (80-120%)		p	Kaolin-kephalin time (21.1-32sec)		p	Fibrinogen (200-360mg/dl)		p
		Mean	SD		Mean	SD		Mean	SD		Mean	SD	
Cycle 1	1	10.88	0.83	NS	112.26	8.70	NS	25.36	3.10	NS	321.80	413.20	NS
	2	10.99	1.16		110.95	11.27		24.77	2.02		316.21	414.31	

Cycle 2	1	10.86	0.68	NS	111.59	6.73	NS	25.38	2.54	NS	228.10	59.00	NS
	2	10.9	0.97		112.78	7.83		24.98	2.29		218.80	58.41	

Cycle 3	1	11.04	0.67	NS	109.56	6.18	NS	25.54	2.39	NS	245.90	99.43	NS
	2	11.18	0.77		109.06	7.75		25.53	1.87		234.10	62.90	

Cycle 4	1	10.74	0.62	NS	112.79	6.29	NS	25.86	2.25	NS	211.80	50.90	NS
	2	10.91	0.71		111.16	6.80		25.73	2.99		223.00	60.62	

Cycle 5	1	10.79	0.84	NS	112.59	8.45	NS	26.37	2.49	NS	222.11	57.00	NS
	2	10.86	0.47		105.65	24.83		26.07	2.77		202.80	33.81	

NS, nonsignificant.

**Table 4:** The uric acid levels in children and adolescents with Duchenne muscular dystrophy during treatment with granulocyte-colony stimulating factor.

Measurement		Uric acid (2.20-5.60 mg/dl, normal range)		
		Average	SD	P value
Cycle 1	1	4.62	0.83	**<** *0.001*
	2	6.04	1.59	

Cycle 2	1	4.63	0.79	**<** *0.001*
	2	5.69	1.11	

Cycle 3	1	4.7	0.86	**<** *0.001*
	2	6.14	1.66	

Cycle 4	1	4.62	0.66	**<** *0.001*
	2	6.02	1.35	

Cycle 5	1	4.52	0.84	**<** *0.001*
	2	5.89	1.20	

**Table 5:** The aminotransferases activity and the cholesterol level in children and adolescents with Duchenne muscular dystrophy in each cycle of treatment with granulocyte-colony stimulating factor.

Measurement		AST (44 U/l)			ALT (37 U/l)			Cholesterol (120-200 mg/dl)		
		Average	SD	P value	Average	SD	P value	Average	SD	P value
Cycle 1	1	215.05	114.05	*0.001*	224.63	132.88	*0.011*	172.21	38.50	<*0.001*
	2	144.11	87.13		195.37	112.73		143.65	30.81	

Cycle 2	1	147.06	65.28	*0.001*	174.47	101.43	NS	176.24	26.53	<*0.001*
	2	115.35	58.54		166.76	111.36		150.32	29.04	

Cycle 3	1	175.63	110.13	*0.001*	209.63	133.61	*0.006*	171.13	38.92	<*0.001*
	2	110.53	54.54		178.63	123.28		136.40	36.70	

Cycle 4	1	181.16	162.31	NS	224.42	149.88	NS	168.10	33.32	<*0.001*
	2	120.47	87.66		193.00	118.18		140.61	33.30	

Cycle 5	1	168.40	150.10	*0.023*	187.00	141.25	NS	164.62	33.84	<*0.001*
	2	99.33	65.91		167.93	130.43		142.83	35.40	

AST, aspartate transaminase; ALT, alanine transaminase; NS, nonsignificant.

**Table 6:** Glucose levels in children and adolescents with Duchenne muscular dystrophy in each cycle of treatment with granulocyte-colony stimulating factor.

Measurement		Glucose		P value
		Average	SD	
Cycle 1	1	89.59	12.56	NS
	2	85.88	11.81	

Cycle 2	1	86.19	4.13	NS
	2	85.56	8.03	

Cycle 3	1	86.31	8.76	NS
	2	80.63	18.01	

Cycle 4	1	85.12	6.02	*0.039*
	2	79.24	12.70	

Cycle 5	1	84.57	4.50	NS
	2	82.64	5.34	

NS, nonsignificant.

## Data Availability

The study data in excel format used to support the findings of this study have been deposited in the Google drive repository at https://drive.google.com/file/d/1HCvE3qCBeyerqFiWkv-bMcCcAbsrrH0V/view?.
